# A co-crystal of 3-(3,5-dinitro­benzo­yl)-1,1-dimethyl­thio­urea and *N*,*N*-dimethyl-3,5-dinitro­benzamide

**DOI:** 10.1107/S1600536812041864

**Published:** 2012-10-13

**Authors:** Sohail Saeed, Naghmana Rashid, Ray J. Butcher, Sema Öztürk Yildirim, Rizwan Hussain

**Affiliations:** a Department of Chemistry, Research Complex, Allama Iqbal Open University, Islamabad 44000, Pakistan; b Chemistry Department, Howard University, Washington, DC 20059, USA; c Chemistry Department, Howard University, Washington, DC 20059, USA, and, Department of Physics, Faculty of Sciences, Erciyes University, 38039, Kayseri, Turkey; d National Engineering & Scientific Commission, PO Box 2801, Islamabad, Pakistan

## Abstract

In the title compound, C_10_H_10_N_4_O_5_S·C_9_H_9_N_3_O_5_, the amide groups of 3-(3,5-dinitro-benzo­yl)-1,1-dimethyl-thio­urea and *N*,*N*-dimethyl-3,5-dinitro-benzamide mol­ecules are oriented at dihedral angles of 39.13 (8) and 55.97 (11)°, respectively, to the attached benzene rings. In the crystal, the two mol­ecules are linked by an N—H⋯O hydrogen bond. Weak C—H⋯O link the mol­ecules into a sheet parallel to the *bc* plane. C—H⋯S inter­actions also occur.

## Related literature

For related structures, see: Saeed *et al.* (2010*a* ▶,*b* ▶, 2011 ▶, 2012 ▶).
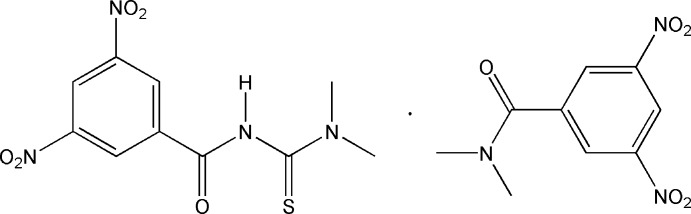


## Experimental

### 

#### Crystal data

C_10_H_10_N_4_O_5_S·C_9_H_9_N_3_O_5_
*M* *_r_* = 537.47
Triclinic, 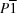

*a* = 9.8457 (5) Å
*b* = 10.0057 (5) Å
*c* = 12.5185 (6) Å
α = 72.413 (5)°
β = 78.428 (4)°
γ = 89.129 (4)°
*V* = 1150.35 (10) Å^3^
*Z* = 2
Cu *K*α radiation
μ = 1.90 mm^−1^
*T* = 123 K
0.44 × 0.38 × 0.27 mm

#### Data collection

Agilent Xcalibur Ruby Gemini diffractometer
Absorption correction: multi-scan (*CrysAlis RED*; Agilent, 2011 ▶) *T*_min_ = 0.488, *T*_max_ = 0.628
7591 measured reflections
4597 independent reflections
4099 reflections with *I* > 2σ(*I*)
*R*_int_ = 0.025

#### Refinement

*R*[*F* ^2^ > 2σ(*F* ^2^)] = 0.041
*wR*(*F* ^2^) = 0.113
*S* = 1.07
4597 reflections
342 parameters
H atoms treated by a mixture of independent and constrained refinement
Δρ_max_ = 0.44 e Å^−3^
Δρ_min_ = −0.33 e Å^−3^

### 

Data collection: *CrysAlis PRO* (Agilent, 2011 ▶); cell refinement: *CrysAlis PRO*; data reduction: *CrysAlis RED* (Agilent, 2011 ▶); program(s) used to solve structure: *SHELXS97* (Sheldrick, 2008 ▶); program(s) used to refine structure: *SHELXL97* (Sheldrick, 2008 ▶); molecular graphics: *SHELXTL* (Sheldrick, 2008 ▶); software used to prepare material for publication: *SHELXTL*.

## Supplementary Material

Crystal structure: contains datablock(s) I, global. DOI: 10.1107/S1600536812041864/xu5627sup1.cif

Structure factors: contains datablock(s) I. DOI: 10.1107/S1600536812041864/xu5627Isup2.hkl

Supplementary material file. DOI: 10.1107/S1600536812041864/xu5627Isup3.cml

Additional supplementary materials:  crystallographic information; 3D view; checkCIF report

## Figures and Tables

**Table 1:** Hydrogen-bond geometry (Å, °)

*D*—H⋯*A*	*D*—H	H⋯*A*	*D*⋯*A*	*D*—H⋯*A*
N3*A*—H1*NA*⋯O5*B*	0.84 (2)	2.07 (2)	2.888 (2)	163 (2)
C2*B*—H2*BA*⋯O1*A* ^i^	0.95	2.51	3.390 (2)	155
C4*B*—H4*BA*⋯O3*A* ^ii^	0.95	2.35	3.163 (2)	143
C6*B*—H6*BA*⋯S1*A*	0.95	2.76	3.6856 (16)	166
C9*A*—H9*AB*⋯O5*B* ^iii^	0.98	2.48	3.368 (2)	150
C9*B*—H9*BB*⋯O4*B* ^iv^	0.98	2.46	3.439 (2)	175
C10*A*—H10*B*⋯O2*A* ^v^	0.98	2.51	3.334 (2)	142

Symmetry codes: (i) 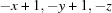; (ii) 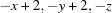; (iii) 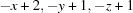; (iv) 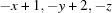; (v) 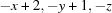.

**Fig. 1.**
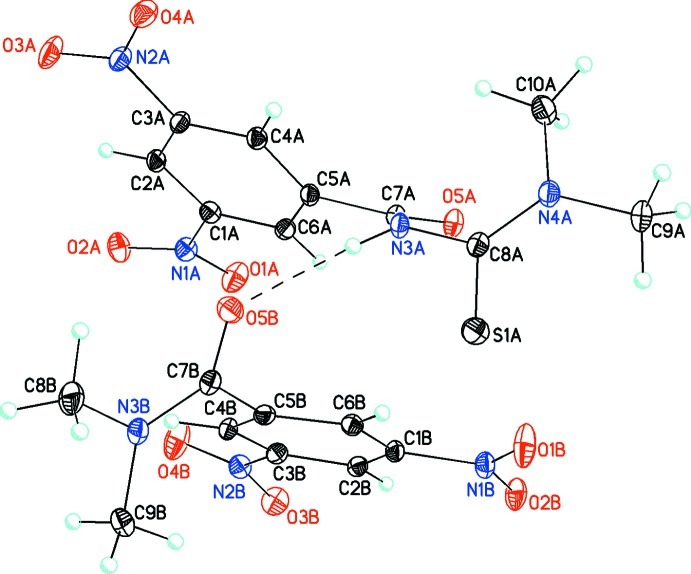
Molecular structure of the title compound, showing the atom-numbering scheme and 30% probability ellipsoids. Intramolecular N—H···O hydrogen bond is indicated by a dashed line.

**Fig. 2.**
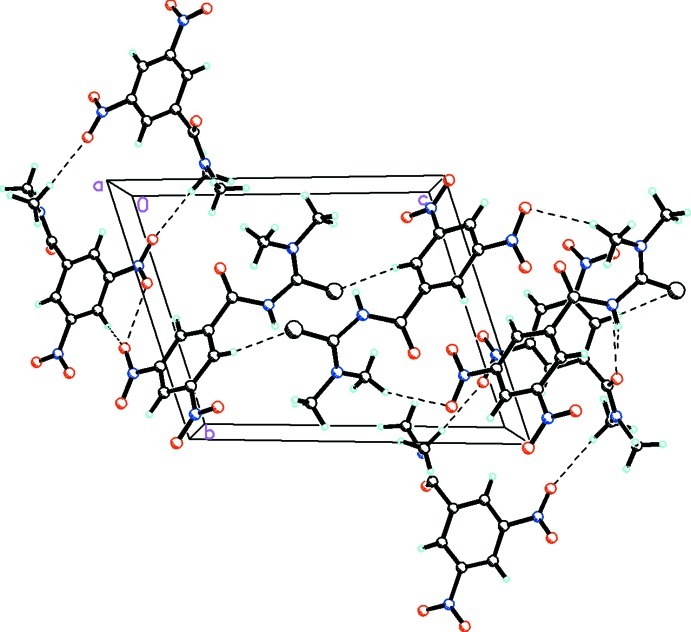
Part of the packing diagram of the title compound, showing a molecular sheet formed by intermolecular N—H···O and C—H···O hydrogen bonds (dashed lines).

## supplementary crystallographic information

### Comment

The crystal structure of the 1:1 adduct of 3-(3,5-dinitro-benzoyl)-1,1-dimethyl-thiourea and N,N-dimethyl-3,5-dinitro-benzamide is reported. It is related to our previous studies on the structural chemistry of heterocyclic compounds containing an N-substituted thiourea (Saeed *et al.*, 2010*a*, 2010*b*, 2011) and amide (Saeed *et al.*, 2012). Herein, as a continuation of these studies, the structure of the title compound, (I), is described.

In the crystal structure of the title compound (Fig. 1), C_10_H_10_N_4_O_5_S, C_9_H_9_N_3_O_5_, there are independent different molecules 3-(3,5-dinitro-benzoyl)-1,1-dimethyl-thiourea(A) and N, *N*-dimethyl-3,5-dinitro-benzamide(B) in the asymmetric unit. Both of the molecule the dinitro-benzene ring systems are planar, with a maximum deviation of 0.295 (1) Å for the O1A atom and 0.286 (2) Å for the O4B atom. In the molecular conformation of 3-(3,5-dinitro-benzoyl)-1,1-dimethyl-thiourea's the C7A=O5A and C8A=S1A bonds are anti to each other. The dihedral angle between the dinitro-benzene unit (C1A—C6A/N1A/N2A/O1A—O4A atoms) and thiourea group (N3A/C8A/N4A/S1A atoms) is 88.2 (1)°. In *N*-dimethyl-3,5-dinitro-benzamide, the dimethyl amide group is rotated by 59.8 (0.1)° out of the plane of the benzene ring.

The 3-(3,5-dinitro-benzoyl)-1,1-dimethyl-thiourea and *N*,*N*-dimethyl-3,5-dinitro-benzamide molecular structure is stabilized by intra- and inter molecular N—H···O and C—H···O hydrogen bonds (Fig. 1 and Table 1). The intermolecular C—H···O hydrogen bonds link the molecules into a sheet parallel to the *bc* plane (Fig. 2).

### Experimental

To a 250 ml round flask fitted with a condenser was added dimethyl amine (0.01 mol), dichloromethane (15 ml) and triethylamine(0.5 ml) with magnetic stirring. 3,5-Dinitrobenzoyl chloride (0.01 mol) was added gradually. The reaction mixture was stirred at room temperature for 1 h and then refluxed for 1.5 h. The product precipitated as a colorless powder, which was washed three times with water and dichloromethane. Recrystallization from ethanol produced the crystals of the title compound.

### Refinement

The H atoms were placed at calculated positions and allowed to ride on their carrier atoms with C—H = 0.95–0.98 Å, and with *U*_iso_ = 1.2–1.5*U*_eq_(C). The N-bound H atom was located in a difference Fourier map and refined freely [refined distances = 0.84 (2) Å].

### Crystal data

**Table:**

C_10_H_10_N_4_O_5_S·C_9_H_9_N_3_O_5_	*Z* = 2
*M**_r_* = 537.47	*F*(000) = 556
Triclinic, *P*1	*D*_x_ = 1.552 Mg m^−^^3^
Hall symbol: -P 1	Cu *K*α radiation, λ = 1.54184 Å
*a* = 9.8457 (5) Å	Cell parameters from 4858 reflections
*b* = 10.0057 (5) Å	θ = 3.7–75.6°
*c* = 12.5185 (6) Å	µ = 1.90 mm^−^^1^
α = 72.413 (5)°	*T* = 123 K
β = 78.428 (4)°	Prism, colorless
γ = 89.129 (4)°	0.44 × 0.38 × 0.27 mm
*V* = 1150.35 (10) Å^3^	

### Data collection

**Table:**

Agilent Xcalibur Ruby Gemini diffractometer	4597 independent reflections
Radiation source: Enhance (Cu) X-ray Source	4099 reflections with *I* > 2σ(*I*)
Graphite monochromator	*R*_int_ = 0.025
Detector resolution: 10.5081 pixels mm^-1^	θ_max_ = 75.7°, θ_min_ = 3.8°
ω scans	*h* = −8→12
Absorption correction: multi-scan (*CrysAlis RED*; Agilent, 2011)	*k* = −11→12
*T*_min_ = 0.488, *T*_max_ = 0.628	*l* = −15→15
7591 measured reflections	

### Refinement

**Table:**

Refinement on *F*^2^	Primary atom site location: structure-invariant direct methods
Least-squares matrix: full	Secondary atom site location: difference Fourier map
*R*[*F*^2^ > 2σ(*F*^2^)] = 0.041	Hydrogen site location: inferred from neighbouring sites
*wR*(*F*^2^) = 0.113	H atoms treated by a mixture of independent and constrained refinement
*S* = 1.07	*w* = 1/[σ^2^(*F*_o_^2^) + (0.0651*P*)^2^ + 0.2475*P*] where *P* = (*F*_o_^2^ + 2*F*_c_^2^)/3
4597 reflections	(Δ/σ)_max_ = 0.001
342 parameters	Δρ_max_ = 0.44 e Å^−^^3^
0 restraints	Δρ_min_ = −0.33 e Å^−^^3^

### Special details

**Table:**

Geometry. All e.s.d.'s (except the e.s.d. in the dihedral angle between two l.s. planes) are estimated using the full covariance matrix. The cell e.s.d.'s are taken into account individually in the estimation of e.s.d.'s in distances, angles and torsion angles; correlations between e.s.d.'s in cell parameters are only used when they are defined by crystal symmetry. An approximate (isotropic) treatment of cell e.s.d.'s is used for estimating e.s.d.'s involving l.s. planes.
Refinement. Refinement of *F*^2^ against ALL reflections. The weighted *R*-factor *wR* and goodness of fit *S* are based on *F*^2^, conventional *R*-factors *R* are based on *F*, with *F* set to zero for negative *F*^2^. The threshold expression of *F*^2^ > σ(*F*^2^) is used only for calculating *R*-factors(gt) *etc*. and is not relevant to the choice of reflections for refinement. *R*-factors based on *F*^2^ are statistically about twice as large as those based on *F*, and *R*- factors based on ALL data will be even larger.

### Fractional atomic coordinates and isotropic or equivalent isotropic displacement parameters (Å^2^)

**Table:**

	*x*	*y*	*z*	*U*_iso_*/*U*_eq_	
S1A	0.76619 (4)	0.42766 (5)	0.57712 (3)	0.02502 (12)	
O1A	0.78961 (16)	0.65356 (16)	−0.12724 (12)	0.0403 (4)	
O2A	0.86851 (15)	0.87002 (14)	−0.18650 (11)	0.0335 (3)	
O3A	1.22867 (15)	0.99829 (15)	−0.02521 (12)	0.0390 (3)	
O4A	1.27475 (14)	0.85999 (14)	0.13226 (11)	0.0310 (3)	
O5A	0.88749 (13)	0.33920 (12)	0.25463 (10)	0.0266 (3)	
O1B	0.53210 (19)	0.27963 (15)	0.46302 (12)	0.0459 (4)	
O2B	0.40878 (15)	0.24042 (13)	0.35063 (12)	0.0352 (3)	
O3B	0.37221 (15)	0.60717 (15)	0.00223 (11)	0.0357 (3)	
O4B	0.5129 (2)	0.78858 (17)	−0.05161 (12)	0.0497 (4)	
O5B	0.81001 (13)	0.76125 (13)	0.32225 (12)	0.0290 (3)	
N1A	0.85641 (16)	0.75121 (16)	−0.12002 (12)	0.0260 (3)	
N2A	1.20846 (15)	0.89025 (15)	0.05610 (12)	0.0253 (3)	
N3A	0.91130 (14)	0.48145 (14)	0.36539 (11)	0.0199 (3)	
N4A	0.94610 (15)	0.26566 (14)	0.49228 (12)	0.0234 (3)	
N1B	0.48356 (16)	0.31620 (15)	0.37681 (12)	0.0254 (3)	
N2B	0.45895 (16)	0.68028 (16)	0.01822 (12)	0.0260 (3)	
N3B	0.63630 (15)	0.91321 (14)	0.30300 (12)	0.0228 (3)	
C1A	0.92940 (17)	0.72182 (17)	−0.02373 (13)	0.0212 (3)	
C2A	1.03083 (17)	0.81896 (17)	−0.02984 (13)	0.0217 (3)	
H2AA	1.0543	0.9010	−0.0937	0.026*	
C3A	1.09632 (16)	0.79080 (17)	0.06156 (14)	0.0205 (3)	
C4A	1.06124 (16)	0.67505 (16)	0.15829 (13)	0.0195 (3)	
H4AA	1.1067	0.6607	0.2207	0.023*	
C5A	0.95709 (16)	0.58035 (16)	0.16093 (13)	0.0189 (3)	
C6A	0.89257 (16)	0.60135 (17)	0.06793 (13)	0.0204 (3)	
H6AA	0.8251	0.5346	0.0676	0.024*	
C7A	0.91461 (16)	0.45272 (16)	0.26354 (13)	0.0198 (3)	
C8A	0.87935 (17)	0.38376 (17)	0.47537 (13)	0.0202 (3)	
C9A	0.9057 (2)	0.14979 (18)	0.59838 (15)	0.0289 (4)	
H9AA	0.8141	0.1653	0.6395	0.043*	
H9AB	0.9740	0.1450	0.6465	0.043*	
H9AC	0.9020	0.0614	0.5803	0.043*	
C10A	1.0716 (2)	0.24386 (19)	0.41521 (15)	0.0301 (4)	
H10A	1.1132	0.3349	0.3641	0.045*	
H10B	1.0474	0.1871	0.3696	0.045*	
H10C	1.1382	0.1950	0.4604	0.045*	
C1B	0.51644 (17)	0.46087 (16)	0.29998 (13)	0.0197 (3)	
C2B	0.47036 (16)	0.49825 (17)	0.19815 (13)	0.0196 (3)	
H2BA	0.4193	0.4340	0.1770	0.024*	
C3B	0.50307 (17)	0.63468 (17)	0.12917 (13)	0.0206 (3)	
C4B	0.57312 (17)	0.73238 (17)	0.15966 (14)	0.0210 (3)	
H4BA	0.5926	0.8257	0.1100	0.025*	
C5B	0.61429 (16)	0.69075 (17)	0.26466 (14)	0.0196 (3)	
C6B	0.58805 (16)	0.55288 (17)	0.33527 (13)	0.0193 (3)	
H6BA	0.6183	0.5224	0.4058	0.023*	
C7B	0.69378 (17)	0.79193 (17)	0.30051 (13)	0.0209 (3)	
C8B	0.7131 (2)	1.01915 (19)	0.32817 (18)	0.0333 (4)	
H8BA	0.8121	1.0004	0.3159	0.050*	
H8BB	0.6795	1.0162	0.4081	0.050*	
H8BC	0.6996	1.1122	0.2775	0.050*	
C9B	0.49061 (19)	0.94183 (18)	0.29843 (15)	0.0278 (4)	
H9BA	0.4410	0.8558	0.3022	0.042*	
H9BB	0.4859	1.0147	0.2267	0.042*	
H9BC	0.4476	0.9741	0.3634	0.042*	
H1NA	0.889 (2)	0.563 (2)	0.3648 (17)	0.021 (5)*	

### Atomic displacement parameters (Å^2^)

**Table:**

	*U*^11^	*U*^22^	*U*^33^	*U*^12^	*U*^13^	*U*^23^
S1A	0.0265 (2)	0.0292 (2)	0.0174 (2)	0.00437 (16)	−0.00275 (15)	−0.00549 (16)
O1A	0.0447 (8)	0.0425 (8)	0.0328 (7)	−0.0136 (6)	−0.0189 (6)	−0.0022 (6)
O2A	0.0478 (8)	0.0284 (7)	0.0226 (6)	0.0057 (6)	−0.0134 (6)	−0.0014 (5)
O3A	0.0428 (8)	0.0286 (7)	0.0343 (7)	−0.0168 (6)	−0.0083 (6)	0.0080 (6)
O4A	0.0298 (7)	0.0310 (7)	0.0315 (7)	−0.0068 (5)	−0.0105 (5)	−0.0053 (5)
O5A	0.0364 (7)	0.0172 (6)	0.0253 (6)	−0.0046 (5)	−0.0065 (5)	−0.0044 (5)
O1B	0.0758 (11)	0.0262 (7)	0.0330 (8)	−0.0100 (7)	−0.0259 (7)	0.0051 (6)
O2B	0.0455 (8)	0.0176 (6)	0.0421 (8)	−0.0077 (5)	−0.0112 (6)	−0.0067 (6)
O3B	0.0376 (8)	0.0423 (8)	0.0311 (7)	−0.0024 (6)	−0.0168 (6)	−0.0105 (6)
O4B	0.0754 (12)	0.0413 (9)	0.0241 (7)	−0.0166 (8)	−0.0160 (7)	0.0066 (6)
O5B	0.0251 (6)	0.0239 (6)	0.0426 (7)	0.0021 (5)	−0.0121 (5)	−0.0138 (5)
N1A	0.0293 (8)	0.0276 (8)	0.0197 (7)	0.0010 (6)	−0.0062 (6)	−0.0043 (6)
N2A	0.0241 (7)	0.0215 (7)	0.0262 (7)	−0.0050 (6)	−0.0010 (6)	−0.0037 (6)
N3A	0.0250 (7)	0.0141 (6)	0.0175 (6)	0.0002 (5)	−0.0013 (5)	−0.0023 (5)
N4A	0.0270 (7)	0.0185 (7)	0.0213 (7)	0.0010 (5)	−0.0025 (5)	−0.0028 (6)
N1B	0.0330 (8)	0.0166 (7)	0.0243 (7)	0.0006 (6)	−0.0030 (6)	−0.0049 (6)
N2B	0.0307 (8)	0.0267 (8)	0.0196 (7)	0.0030 (6)	−0.0050 (6)	−0.0061 (6)
N3B	0.0260 (7)	0.0169 (6)	0.0249 (7)	−0.0019 (5)	−0.0044 (5)	−0.0059 (5)
C1A	0.0234 (8)	0.0225 (8)	0.0173 (7)	0.0031 (6)	−0.0045 (6)	−0.0054 (6)
C2A	0.0242 (8)	0.0180 (8)	0.0178 (7)	0.0015 (6)	0.0002 (6)	−0.0009 (6)
C3A	0.0195 (8)	0.0177 (7)	0.0220 (8)	−0.0024 (6)	−0.0008 (6)	−0.0049 (6)
C4A	0.0212 (8)	0.0181 (7)	0.0177 (7)	0.0013 (6)	−0.0031 (6)	−0.0036 (6)
C5A	0.0214 (8)	0.0156 (7)	0.0172 (7)	0.0015 (6)	−0.0006 (6)	−0.0037 (6)
C6A	0.0201 (8)	0.0196 (8)	0.0208 (8)	−0.0004 (6)	−0.0025 (6)	−0.0064 (6)
C7A	0.0202 (8)	0.0162 (7)	0.0201 (8)	−0.0005 (6)	−0.0023 (6)	−0.0025 (6)
C8A	0.0217 (8)	0.0191 (8)	0.0184 (7)	−0.0021 (6)	−0.0043 (6)	−0.0034 (6)
C9A	0.0355 (10)	0.0188 (8)	0.0264 (9)	−0.0012 (7)	−0.0054 (7)	0.0012 (7)
C10A	0.0352 (10)	0.0261 (9)	0.0259 (9)	0.0083 (7)	−0.0038 (7)	−0.0054 (7)
C1B	0.0214 (8)	0.0151 (7)	0.0209 (8)	0.0005 (6)	−0.0012 (6)	−0.0052 (6)
C2B	0.0199 (7)	0.0184 (7)	0.0213 (8)	−0.0014 (6)	−0.0017 (6)	−0.0086 (6)
C3B	0.0215 (8)	0.0224 (8)	0.0174 (7)	0.0004 (6)	−0.0027 (6)	−0.0061 (6)
C4B	0.0217 (8)	0.0166 (7)	0.0212 (8)	−0.0015 (6)	−0.0009 (6)	−0.0027 (6)
C5B	0.0170 (7)	0.0191 (8)	0.0224 (8)	0.0004 (6)	−0.0010 (6)	−0.0080 (6)
C6B	0.0195 (7)	0.0192 (8)	0.0195 (7)	0.0017 (6)	−0.0032 (6)	−0.0067 (6)
C7B	0.0232 (8)	0.0179 (7)	0.0205 (7)	−0.0030 (6)	−0.0027 (6)	−0.0052 (6)
C8B	0.0383 (10)	0.0214 (9)	0.0427 (11)	−0.0042 (7)	−0.0063 (8)	−0.0144 (8)
C9B	0.0321 (9)	0.0213 (8)	0.0295 (9)	0.0072 (7)	−0.0088 (7)	−0.0058 (7)

### Geometric parameters (Å, º)

**Table:**

S1A—C8A	1.6764 (16)	C3A—C4A	1.387 (2)
O1A—N1A	1.221 (2)	C4A—C5A	1.396 (2)
O2A—N1A	1.219 (2)	C4A—H4AA	0.9500
O3A—N2A	1.227 (2)	C5A—C6A	1.396 (2)
O4A—N2A	1.2230 (19)	C5A—C7A	1.505 (2)
O5A—C7A	1.213 (2)	C6A—H6AA	0.9500
O1B—N1B	1.221 (2)	C9A—H9AA	0.9800
O2B—N1B	1.220 (2)	C9A—H9AB	0.9800
O3B—N2B	1.217 (2)	C9A—H9AC	0.9800
O4B—N2B	1.216 (2)	C10A—H10A	0.9800
O5B—C7B	1.242 (2)	C10A—H10B	0.9800
N1A—C1A	1.477 (2)	C10A—H10C	0.9800
N2A—C3A	1.475 (2)	C1B—C2B	1.382 (2)
N3A—C7A	1.383 (2)	C1B—C6B	1.389 (2)
N3A—C8A	1.404 (2)	C2B—C3B	1.379 (2)
N3A—H1NA	0.84 (2)	C2B—H2BA	0.9500
N4A—C8A	1.324 (2)	C3B—C4B	1.389 (2)
N4A—C9A	1.462 (2)	C4B—C5B	1.394 (2)
N4A—C10A	1.465 (2)	C4B—H4BA	0.9500
N1B—C1B	1.474 (2)	C5B—C6B	1.391 (2)
N2B—C3B	1.476 (2)	C5B—C7B	1.509 (2)
N3B—C7B	1.337 (2)	C6B—H6BA	0.9500
N3B—C8B	1.455 (2)	C8B—H8BA	0.9800
N3B—C9B	1.468 (2)	C8B—H8BB	0.9800
C1A—C2A	1.379 (2)	C8B—H8BC	0.9800
C1A—C6A	1.383 (2)	C9B—H9BA	0.9800
C2A—C3A	1.378 (2)	C9B—H9BB	0.9800
C2A—H2AA	0.9500	C9B—H9BC	0.9800
			
O2A—N1A—O1A	125.14 (15)	N4A—C9A—H9AA	109.5
O2A—N1A—C1A	117.76 (14)	N4A—C9A—H9AB	109.5
O1A—N1A—C1A	117.09 (14)	H9AA—C9A—H9AB	109.5
O4A—N2A—O3A	124.45 (15)	N4A—C9A—H9AC	109.5
O4A—N2A—C3A	118.26 (14)	H9AA—C9A—H9AC	109.5
O3A—N2A—C3A	117.29 (14)	H9AB—C9A—H9AC	109.5
C7A—N3A—C8A	125.83 (14)	N4A—C10A—H10A	109.5
C7A—N3A—H1NA	114.8 (13)	N4A—C10A—H10B	109.5
C8A—N3A—H1NA	112.8 (13)	H10A—C10A—H10B	109.5
C8A—N4A—C9A	120.93 (14)	N4A—C10A—H10C	109.5
C8A—N4A—C10A	124.44 (14)	H10A—C10A—H10C	109.5
C9A—N4A—C10A	114.36 (14)	H10B—C10A—H10C	109.5
O2B—N1B—O1B	123.94 (15)	C2B—C1B—C6B	123.74 (15)
O2B—N1B—C1B	117.94 (14)	C2B—C1B—N1B	117.83 (14)
O1B—N1B—C1B	118.12 (15)	C6B—C1B—N1B	118.40 (14)
O4B—N2B—O3B	124.34 (15)	C3B—C2B—C1B	115.83 (15)
O4B—N2B—C3B	117.45 (15)	C3B—C2B—H2BA	122.1
O3B—N2B—C3B	118.21 (14)	C1B—C2B—H2BA	122.1
C7B—N3B—C8B	119.81 (15)	C2B—C3B—C4B	123.37 (15)
C7B—N3B—C9B	124.36 (14)	C2B—C3B—N2B	118.42 (14)
C8B—N3B—C9B	115.22 (14)	C4B—C3B—N2B	118.19 (14)
C2A—C1A—C6A	123.23 (15)	C3B—C4B—C5B	118.68 (15)
C2A—C1A—N1A	117.38 (14)	C3B—C4B—H4BA	120.7
C6A—C1A—N1A	119.38 (15)	C5B—C4B—H4BA	120.7
C3A—C2A—C1A	116.55 (15)	C6B—C5B—C4B	120.01 (15)
C3A—C2A—H2AA	121.7	C6B—C5B—C7B	119.19 (14)
C1A—C2A—H2AA	121.7	C4B—C5B—C7B	120.73 (14)
C2A—C3A—C4A	123.43 (15)	C1B—C6B—C5B	118.31 (15)
C2A—C3A—N2A	117.94 (14)	C1B—C6B—H6BA	120.8
C4A—C3A—N2A	118.63 (14)	C5B—C6B—H6BA	120.8
C3A—C4A—C5A	117.88 (15)	O5B—C7B—N3B	123.42 (15)
C3A—C4A—H4AA	121.1	O5B—C7B—C5B	118.99 (14)
C5A—C4A—H4AA	121.1	N3B—C7B—C5B	117.54 (14)
C4A—C5A—C6A	120.56 (14)	N3B—C8B—H8BA	109.5
C4A—C5A—C7A	120.30 (14)	N3B—C8B—H8BB	109.5
C6A—C5A—C7A	119.12 (14)	H8BA—C8B—H8BB	109.5
C1A—C6A—C5A	118.24 (15)	N3B—C8B—H8BC	109.5
C1A—C6A—H6AA	120.9	H8BA—C8B—H8BC	109.5
C5A—C6A—H6AA	120.9	H8BB—C8B—H8BC	109.5
O5A—C7A—N3A	125.54 (15)	N3B—C9B—H9BA	109.5
O5A—C7A—C5A	122.24 (14)	N3B—C9B—H9BB	109.5
N3A—C7A—C5A	112.22 (13)	H9BA—C9B—H9BB	109.5
N4A—C8A—N3A	117.18 (14)	N3B—C9B—H9BC	109.5
N4A—C8A—S1A	124.68 (12)	H9BA—C9B—H9BC	109.5
N3A—C8A—S1A	118.07 (12)	H9BB—C9B—H9BC	109.5
			
O2A—N1A—C1A—C2A	−14.8 (2)	C7A—N3A—C8A—N4A	50.1 (2)
O1A—N1A—C1A—C2A	164.50 (16)	C7A—N3A—C8A—S1A	−132.89 (15)
O2A—N1A—C1A—C6A	164.30 (15)	O2B—N1B—C1B—C2B	−5.4 (2)
O1A—N1A—C1A—C6A	−16.4 (2)	O1B—N1B—C1B—C2B	175.00 (16)
C6A—C1A—C2A—C3A	0.0 (2)	O2B—N1B—C1B—C6B	172.73 (15)
N1A—C1A—C2A—C3A	179.05 (14)	O1B—N1B—C1B—C6B	−6.9 (2)
C1A—C2A—C3A—C4A	−2.6 (2)	C6B—C1B—C2B—C3B	1.8 (2)
C1A—C2A—C3A—N2A	178.07 (14)	N1B—C1B—C2B—C3B	179.79 (13)
O4A—N2A—C3A—C2A	−173.97 (15)	C1B—C2B—C3B—C4B	−2.4 (2)
O3A—N2A—C3A—C2A	6.0 (2)	C1B—C2B—C3B—N2B	178.90 (14)
O4A—N2A—C3A—C4A	6.7 (2)	O4B—N2B—C3B—C2B	−164.52 (17)
O3A—N2A—C3A—C4A	−173.32 (16)	O3B—N2B—C3B—C2B	15.0 (2)
C2A—C3A—C4A—C5A	2.1 (2)	O4B—N2B—C3B—C4B	16.7 (2)
N2A—C3A—C4A—C5A	−178.57 (13)	O3B—N2B—C3B—C4B	−163.76 (16)
C3A—C4A—C5A—C6A	1.0 (2)	C2B—C3B—C4B—C5B	0.8 (2)
C3A—C4A—C5A—C7A	179.95 (14)	N2B—C3B—C4B—C5B	179.49 (14)
C2A—C1A—C6A—C5A	2.9 (2)	C3B—C4B—C5B—C6B	1.6 (2)
N1A—C1A—C6A—C5A	−176.12 (14)	C3B—C4B—C5B—C7B	178.49 (14)
C4A—C5A—C6A—C1A	−3.4 (2)	C2B—C1B—C6B—C5B	0.4 (2)
C7A—C5A—C6A—C1A	177.66 (14)	N1B—C1B—C6B—C5B	−177.57 (14)
C8A—N3A—C7A—O5A	1.6 (3)	C4B—C5B—C6B—C1B	−2.1 (2)
C8A—N3A—C7A—C5A	−178.18 (14)	C7B—C5B—C6B—C1B	−179.10 (14)
C4A—C5A—C7A—O5A	−140.51 (17)	C8B—N3B—C7B—O5B	2.3 (3)
C6A—C5A—C7A—O5A	38.5 (2)	C9B—N3B—C7B—O5B	−168.35 (16)
C4A—C5A—C7A—N3A	39.3 (2)	C8B—N3B—C7B—C5B	−175.18 (15)
C6A—C5A—C7A—N3A	−141.73 (15)	C9B—N3B—C7B—C5B	14.2 (2)
C9A—N4A—C8A—N3A	−171.11 (15)	C6B—C5B—C7B—O5B	55.5 (2)
C10A—N4A—C8A—N3A	15.3 (2)	C4B—C5B—C7B—O5B	−121.47 (17)
C9A—N4A—C8A—S1A	12.1 (2)	C6B—C5B—C7B—N3B	−126.92 (16)
C10A—N4A—C8A—S1A	−161.53 (14)	C4B—C5B—C7B—N3B	56.1 (2)

### Hydrogen-bond geometry (Å, º)

**Table:**

*D*—H···*A*	*D*—H	H···*A*	*D*···*A*	*D*—H···*A*
N3*A*—H1*NA*···O5*B*	0.84 (2)	2.07 (2)	2.888 (2)	163 (2)
C2*B*—H2*BA*···O1*A*^i^	0.95	2.51	3.390 (2)	155
C4*B*—H4*BA*···O3*A*^ii^	0.95	2.35	3.163 (2)	143
C6*B*—H6*BA*···S1*A*	0.95	2.76	3.6856 (16)	166
C9*A*—H9*AB*···O5*B*^iii^	0.98	2.48	3.368 (2)	150
C9*B*—H9*BB*···O4*B*^iv^	0.98	2.46	3.439 (2)	175
C10*A*—H10*B*···O2*A*^v^	0.98	2.51	3.334 (2)	142

Symmetry codes: (i) −*x*+1, −*y*+1, −*z*; (ii) −*x*+2, −*y*+2, −*z*; (iii) −*x*+2, −*y*+1, −*z*+1; (iv) −*x*+1, −*y*+2, −*z*; (v) −*x*+2, −*y*+1, −*z*.
